# Celebrating women in physiology: Marie Krogh and the single‐breath technique for measuring pulmonary diffusing capacity

**DOI:** 10.1113/EP092377

**Published:** 2024-11-19

**Authors:** Ronan M. G. Berg

**Affiliations:** ^1^ Centre for Physical Activity Research Copenhagen University Hospital – Rigshospitalet Copenhagen Denmark; ^2^ Department of Clinical Physiology and Nuclear Medicine Copenhagen University Hospital – Rigshospitalet Copenhagen Denmark; ^3^ Department of Biomedical Sciences, Faculty of Health and Medical Sciences University of Copenhagen Copenhagen Denmark; ^4^ Neurovascular Research Laboratory, Faculty of Life Sciences and Education University of South Wales Pontypridd UK

On 25 December 1874, in the parish of Husby, Denmark, Marie Krogh (1874–1943; born Birte Marie Jørgensen) was born. This editorial in the December issue of *Experimental Physiology*, published on the 150th anniversary of her birth, is dedicated to celebrating her contributions to science, and in particular to physiology.

In March 1905, Marie Jørgensen (Figure 1) married August Krogh (1874–1949). He was born one month earlier than her, in Grenaa, Denmark, and received the 1920 Nobel Prize in Physiology or Medicine for his work on capillary physiology (Krogh, 1919a, 1919b, 1919c). August Krogh was undoubtedly a genius and probably the most influential Danish scientist within biomedical sciences to date, but I often find that Marie Krogh's contributions are somewhat overlooked. Not only did she manage their household, she also continuously mediated conflicts and tensions arising from August Krogh's stubbornness and brusqueness, which often caused friction with friends, relatives and collaborators, enabling him to pursue his various ideas without worry (Sindbæk, 2022). Marie Krogh also served as his sparring partner, discussing their data and ideas for hours in the evenings. As a physician, she worked as a full‐time clinician, but nonetheless concurrently pursued her own line of research. In fact, she was one of the most prominent Danish scientists of her time, having a tremendous impact through her research in diverse areas such as respiratory physiology, endocrinology, pharmacology and nutrition. A full account of her research portfolio is beyond the scope of this editorial. Here, I would like to pay homage to her development of the single‐breath technique for measuring pulmonary diffusing capacity, published in *The Journal of Physiology* (Krogh, 1915). The historical importance of this technique has also been recognised by others before me (Hughes & Borland, 2015; Morrell, 2015), as it remains in use for both research and clinical purposes to this day.

**FIGURE 1:**
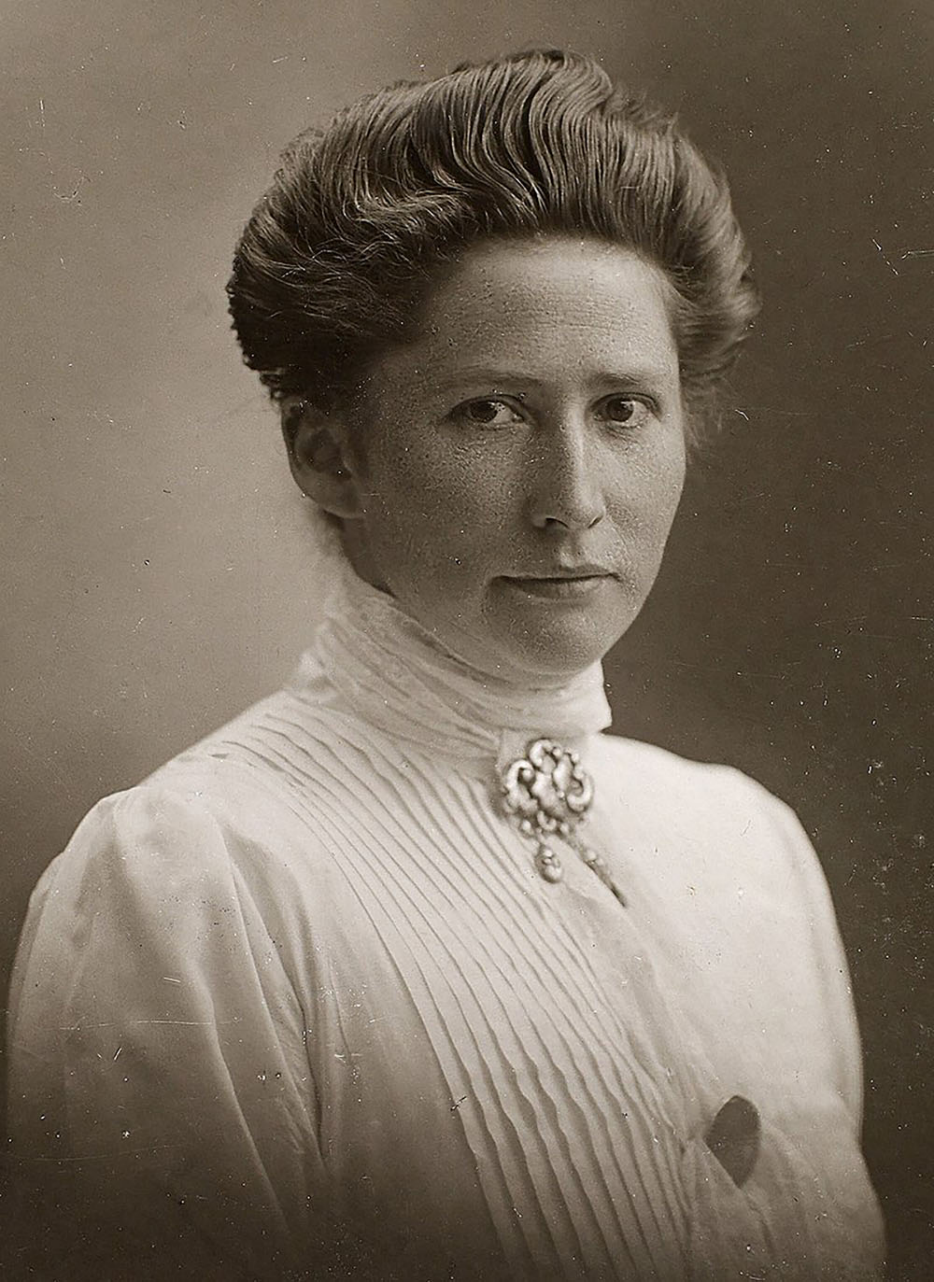
Marie Krogh (1874–1943), MD (1907), DMSc (1914). Photographed ca. 1910. Copyright: public domain.

To me, Marie Krogh had a very practice‐oriented approach to science, viewing it as a means to resolve whatever real‐world problems she encountered. This began while she was still a medical student, at the time when she had just met August Krogh, her instructor at medical school. Deeply in love, they exchanged long and devoted letters, but their joy was overshadowed by tragedy. In July 1904, her sister‐in‐law and close friend, Emma, died in childbirth. Marie was present when Emma gave birth to a healthy boy, and though the birth had initially seemed normal, it became apparent that Emma suffered from an incomplete placental separation –c placenta accreta – one of the most common causes of death in childbirth at the time, for which no treatment options were available. The midwife summoned a village doctor, and Marie sat there for hours with the family knowing that her friend was unlikely to survive. When the doctor arrived and removed the placenta accreta with forceps – clinging to the hope that the consequent blood loss would be minimal – Emma bled to death, at the age of 32. A few days later in a letter to August Krogh, Marie wrote: ‘Dear Friend, you cannot imagine how terrible it is to face this, to see one of your closest die without being able to do anything to save her. It is so cruelly unforgiving to see a young, healthy person, whom we all wanted so much to keep, and who herself was so happy with life and her home, die without anything being done to help her’ (Sindbæk, 2022). It is not surprising, therefore, that the first experiments Marie Krogh set up with August the following spring, while their mentor and head of the laboratory, Professor Christian Bohr (1855–1911), was travelling to England, focused on the treatment of severe haemorrhage. Unfortunately, the laboratory assistant, who handled all practical matters such as generating electricity and producing distilled water, fell ill, so the experiments had to be suspended and were never resumed.

Instead, over the next several years, Marie Krogh, together with August Krogh, began studying pulmonary gas exchange, becoming part of the now legendary controversy over its mechanism. On one side stood their mentor, Christian Bohr, and J. S. Haldane (1860–1936), while the Kroghs were on the other. This controversy could be traced all the way back to the dispute between Carl Ludwig (1816–1895) and Eduard Pflüger (1829–1910), two of the most prominent 19th‐century physiologists who were largely responsible for establishing physiology as an experimental science, both of them viewing life's processes as governed by the laws of physics and chemistry alone, thus requiring analysis through these scientific disciplines – an approach embraced by Bohr, Haldane and the Kroghs alike.

I will refrain from going deeper into this controversy here – excellent detailed accounts can be found elsewhere (Astrup & Severinghaus, 1986; Gjedde, 2010; Schmidt‐Nielsen, 1995) – but I will briefly mention that Bohr, in the prestigious *Handbuch der Physiologie des Menschen* edited by the German physiologist Willibald Nagel (1870–1911), and probably the most authoritative physiology publication at the time, had provided a compelling secretion theory of pulmonary gas exchange (Bohr, 1905). He proposed that, like the swim bladder of some deep‐sea fish, the lungs could secrete gases against normal tension gradients, a theory based on over a decade of experimental research, by which Bohr provided one of the first comprehensive models of respiration, and which earned him two Nobel Prize nominations. However, through diverse experiments on rabbits, tortoises, cattle and humans conducted as part of a series of studies that would later become known as ‘The Seven Little Devils’ (Krogh, 1910a, 1910b, 1910c, 1910d, 1910e; Krogh & Krogh, 1910a, 1910b), the Kroghs systematically disproved the fundamental premises of Bohr's secretion theory. As part of this work, they performed experiments on themselves using an early version of the single‐breath technique in July 1909 (Krogh & Krogh, 1910b). They inhaled a specific quantity of air containing carbon monoxide (CO) and determined the amount absorbed, calculating what they called the ‘diffusion constant’ – now unequivocally known as the pulmonary diffusing capacity for CO (*D*_L,CO_). The same summer, Bohr conducted experiments on himself, measuring *D*_L,CO_ in a similar manner at rest and immediately after stair‐walking to exhaustion, and he reached the opposite conclusion (Bohr, 1909).

After the publication of The Seven Little Devils, August Krogh shifted his focus to collaboration with Johannes Lindhard (1870–1947), conducting measurements on cardiac output and ventilation during exercise, which would mark the birth of Scandinavian exercise physiology (Berg, 2024a, 2024b). Meanwhile, Marie Krogh chose to continue pursuing the mechanisms of pulmonary gas exchange at rest and during exercise. She modified and optimised the protocol for the single‐breath *D*_L,CO_ technique, refining the breathing procedure, lung volume measurements, and CO quantification, as well as calculations for CO uptake (*K*_CO_) during the manoeuvre, and the measurement of alveolar volume (Krogh, 1915). In the protocol, after exhaling to residual volume, the subject inhaled a mixture of CO (1%) in air up to total lung capacity, and after a brief breath‐hold, forcefully expired approximately half of their vital capacity, with the last portion of the exhaled gas being sampled to determine its CO concentration. Over the next few years, she recruited 22 healthy individuals (five females) aged 10–65 years, as well as eight patients from her clinical practice with lung disease. The observant reader will note that Marie Krogh (subject 16), August Krogh (subject 17) and probably also Johannes Lindhard (subject 20) are among the research subjects. In fact, August Krogh had the lowest *K*_CO_ of all participants, which clearly puzzled Marie, to such an extent that she dedicated several paragraphs in the paper to discussing this. She ruled out that this anomaly was genetic by recruiting his two younger brothers, Johan (subject 14) and Emil (subject 15), as well as his father Viggo (subject 20), all of whom showed seemingly normal values. August Krogh had experienced severe pneumonia in childhood, but three other individuals who had suffered from pneumonia within the past 2 months to 2 years also displayed normal values. Thus, August Krogh's low *K*_CO_ remained an unexplained finding that neither she nor anyone else ever managed to resolve.

Marie Krogh's findings demonstrated that *D*_L,CO_ varies with age and body proportions, as assessed by surface area estimations, being greater in men than in women and greater in adults than in children (Krogh, 1915). She also obtained measurements in four individuals with obstructive lung disease and one with tuberculosis, all of whom exhibited relatively low *D*_L,CO_ values. Additionally, she performed measurements during exercise on herself and two other participants using a bicycle ergometer constructed by August Krogh (Krogh, 1913). August Krogh and another participant provided measurements immediately after stair‐walking, closely resembling the experimental set‐up Bohr had originally devised. In all cases, *D*_L,CO_ increased tremendously, by up to 40%. These findings lent further support to the refutation of the secretion theory. Although the controversy over the mechanism of gas exchange persisted until Haldane's death decades later, Marie Krogh eventually shifted her focus to other areas of research. Nevertheless, her work stands out as a seminal contribution, and her measurements in patients had important clinical implications. However, the single‐breath technique was never implemented clinically during her lifetime, mainly due to its laborious nature. The difficulty and time‐consuming process of measuring CO, which was performed using a so‐called modified Petterson apparatus (Petterson, 1886), hindered its broader application.

This all changed when it was realised that techniques developed for gas detection during World War II, including the infrared CO meter, provided much quicker and more practical CO measurements for *D*_L,CO_ assessments. At this time, impaired pulmonary gas exchange was becoming an increasingly recognised problem in patients with lung fibrosis (Baldwin et al., 1949), who exhibited marked arterial desaturation during exercise and also reduced diffusing capacity measured by alternative and much more cumbersome methodology (Austrian et al., 1951). This led to the introduction of the concept of the ‘alveolar–capillary block’, now known as ‘diffusion limitation’ (Austrian et al., 1951). In the 1960s, a series of studies thus revisited Marie Krogh's single‐breath technique, incorporating infrared CO measurements for both inspired and expired air, along with slight modifications to the breathing technique – most notably, exhalation to residual volume rather than half of vital capacity (Forster et al., 1955, 1954; Fowler, 1952). This method was attractive because it allowed the assessment of pulmonary gas exchange without the need for arterial blood gas sampling. Soon, the first standardised guidelines for clinical use were developed (Ogilvie et al., 1957), and as part of this advancement, a mathematical reassessment of the method was published in this journal – then entitled *Quarterly Journal of Experimental Physiology and Cognate Medical Sciences* – demonstrating that the validity and reliability of *D*_L,CO_ measurements depended critically on the breath‐hold time, which was also standardised (Jones & Meade, 1961). Following these developments, *D*_L,CO_ measurement by the single‐breath technique became one of the most widely used methods for lung function assessment, along with dynamic spirometry and body plethysmography, and remains so to this day (Graham et al., 2017), where it is used for diagnostic classification, prognostication and evaluating treatment effects. Even Marie Krogh herself had probably not imagined that!

I recently had the privilege of serving as a scientific consultant to author and journalist Hanne Sindbæk as she wrote an extensive biography in Danish on August and Marie Krogh (Sindbæk, 2022). Upon the book's publication, I was invited to a reception to celebrate its release, where I had the pleasure of meeting the present‐day members of the Krogh family. Among them was August and Marie Krogh's grandson, Lars Wernstedt (1939–2022). Despite being very young when Marie Krogh passed away, he told me that he had the fondest memories of her (Figure 2). Like Marie Krogh, Wernstedt was a physician, and he told me that he had worked within the field of pulmonary medicine in his youth, and had prescribed *D*_L,CO_ measurements for years, as the method became increasingly popular in the 1970s. It was deeply moving to hear him recount how he became emotionally overwhelmed upon one day at work suddenly realising that the measurement he was prescribing and assessing on a daily basis was, in fact, the very method his beloved grandmother had developed.

**FIGURE 2:**
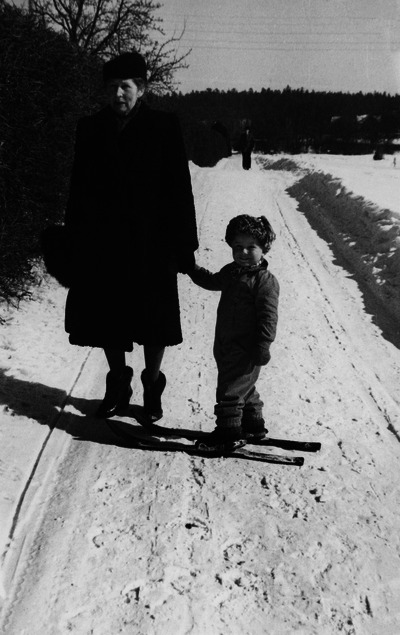
Marie Krogh with her grandchild Lars. Photographed in Stockholm in March 1942. Printed with permission from Hanne Sindbæk.

August Krogh was extremely inventive, and he is probably among the physiologists with the most eponyms attached to his name, including the Krogh microtonometer, the Krogh spirometer, the Krogh ergometer, the Krogh cylinder (or Krogh model), the Krogh–Erlang equation, the Krogh principle, and the Krogh respirator. Then there is *K*_CO_, which is still provided in lung function reports today, albeit in a slightly mathematically modified form (Hughes & Pride, 2012), and which is referred to as the Krogh factor. However, in contrast to what many believe, this does not refer to August, but to Marie Krogh, as it was her concept, first alluded to in their joint paper from 1910 and fully developed in the work that was published in *The Journal of Physiology* in 1915, and which earned her the *doctor medicinae* degree (doctor of medical science; a higher doctoral degree), which had only previously been achieved by three other women before her in Denmark (Sindbæk, 2022). In any event, eponyms and degrees aside, Marie Krogh is a stellar example of The Physiological Society's ‘Women in Physiology’ initiatives (Bailey, 2023; Wray & Stokes, 2013; Wray & Tansey, 2015), an outstanding scientist who still serves as a role model for us all to this day, 150 years after her birth.

## AUTHOR CONTRIBUTIONS

Sole author.

## CONFLICT OF INTEREST

None declared.

## FUNDING INFORMATION

The Centre for Physical Activity Research (CFAS) is supported by TrygFonden (grants ID 101390 and ID 20045). The funders had no role in the decision to prepare, write or publish the manuscript.
